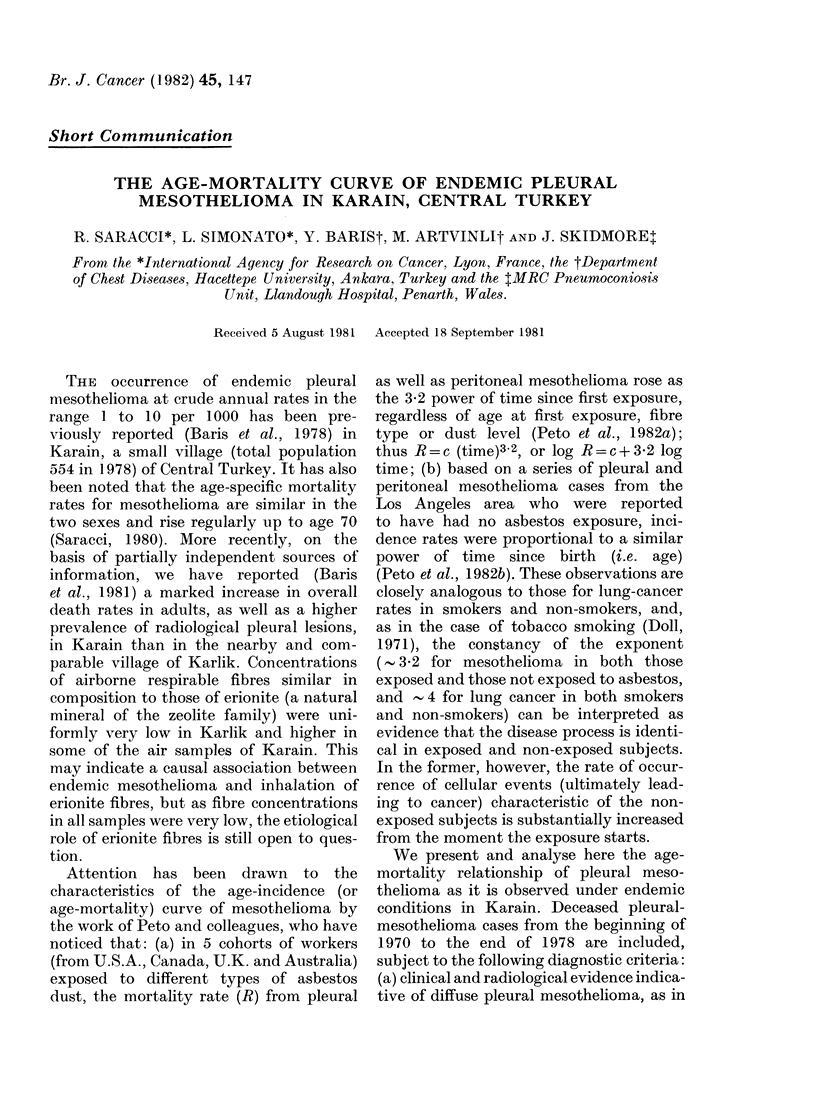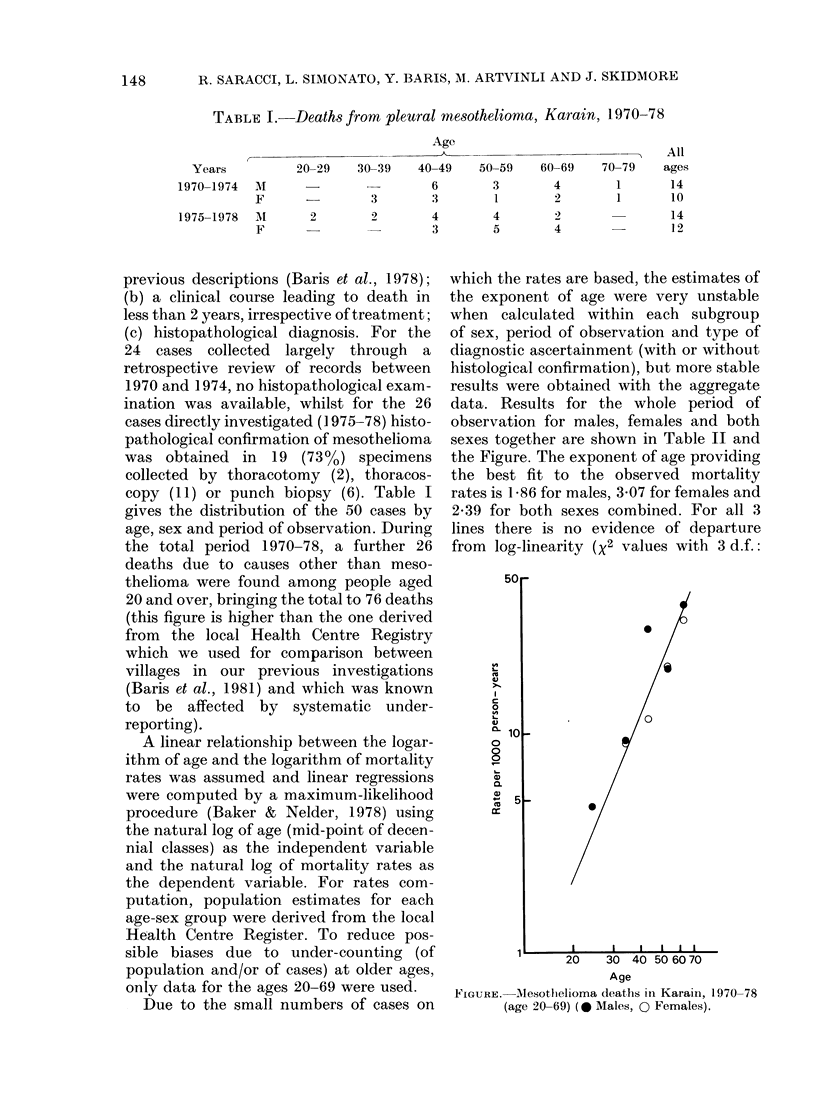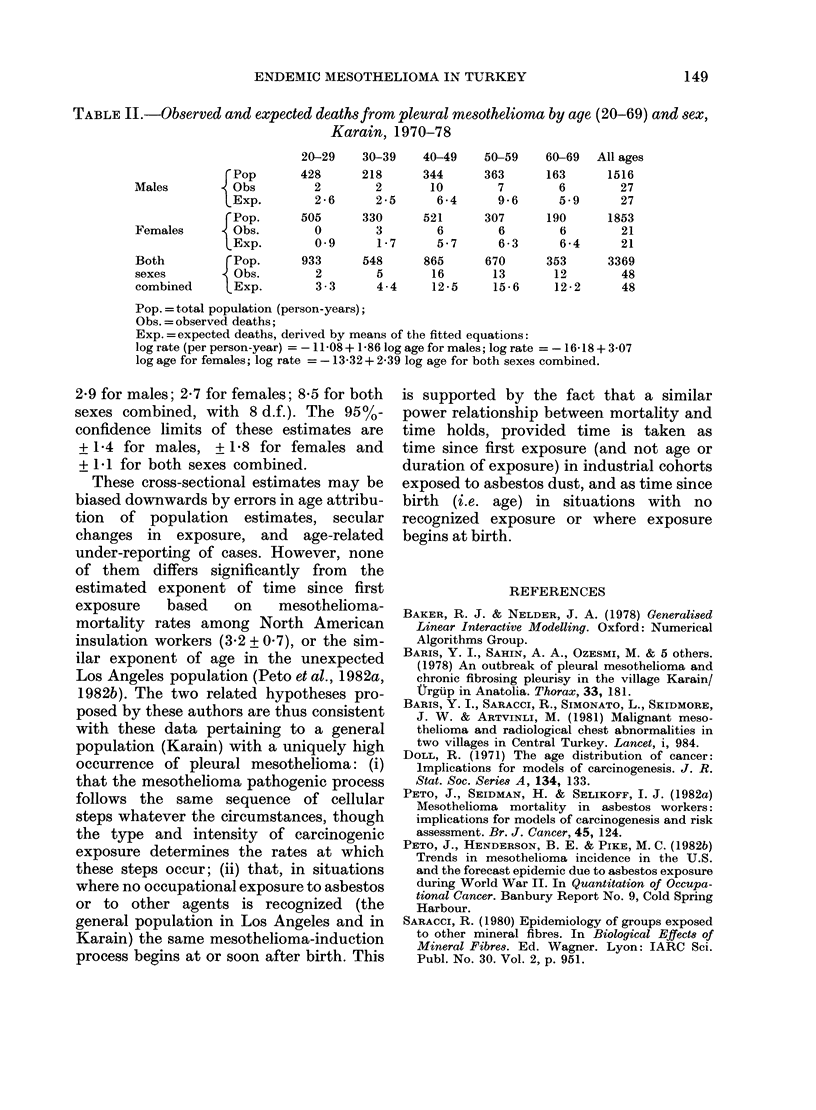# The age-mortality curve of endemic pleural mesothelioma in Karain, Central Turkey.

**DOI:** 10.1038/bjc.1982.19

**Published:** 1982-01

**Authors:** R. Saracci, L. Simonato, Y. Baris, M. Artvinli, J. Skidmore


*Br. J. Cancer* (1982) **45**, 147

***Short Communication***

### THE AGE-MORTALITY CURVE OF ENDEMIC PLEURAL MESOTHELIOMA IN KARAIN, CENTRAL TURKEY

R. SARACCI*, L. SIMONATO*, Y. BARIS†, M. ARTVINLI† AND J. SKIDMORE‡

*From the *International Agency for Research on Cancer, Lyon, France, the †Department of Chest Diseases, Hacettepe University, Ankara, Turkey and the ‡MRC Pneumoconiosis Unit, Llandough Hospital, Penarth, Wales.*

Received 5 August 1981 Accepted 18 September 1981

THE occurrence of endemic pleural mesothelioma at crude annual rates in the range 1 to 10 per 1000 has been previously reported (Baris *et al.*, 1978) in Karain, a small village (total population 554 in 1978) of Central Turkey. It has also been noted that the age-specific mortality rates for mesothelioma are similar in the two sexes and rise regularly up to age 70 (Saracci, 1980). More recently, on the basis of partially independent sources of information, we have reported (Baris *et al.*, 1981) a marked increase in overall death rates in adults, as well as a higher prevalence of radiological pleural lesions, in Karain than in the nearby and comparable village of Karlik. Concentrations of airborne respirable fibres similar in composition to those of erionite (a natural mineral of the zeolite family) were uniformly very low in Karlik and higher in some of the air samples of Karain. This may indicate a causal association between endemic mesothelioma and inhalation of erionite fibres, but as fibre concentrations in all samples were very low, the etiological role of erionite fibres is still open to question.

Attention has been drawn to the characteristics of the age-incidence (or age-mortality) curve of mesothelioma by the work of Peto and colleagues, who have noticed that: (a) in 5 cohorts of workers (from U.S.A., Canada, U.K. and Australia) exposed to different types of asbestos dust, the mortality rate ($R$) from pleural as well as peritoneal mesothelioma rose as the 3·2 power of time since first exposure, regardless of age at first exposure, fibre type or dust level (Peto *et al.*, 1982*a*); thus $R = c\ (\text{time})^{3 \cdot 2}$, or $\log R = c + 3 \cdot 2 \log$ time; (b) based on a series of pleural and peritoneal mesothelioma cases from the Los Angeles area who were reported to have had no asbestos exposure, incidence rates were proportional to a similar power of time since birth (*i.e.* age) (Peto *et al.*, 1982*b*). These observations are closely analogous to those for lung-cancer rates in smokers and non-smokers, and, as in the case of tobacco smoking (Doll, 1971), the constancy of the exponent ($\sim 3 \cdot 2$ for mesothelioma in both those exposed and those not exposed to asbestos, and $\sim 4$ for lung cancer in both smokers and non-smokers) can be interpreted as evidence that the disease process is identical in exposed and non-exposed subjects. In the former, however, the rate of occurrence of cellular events (ultimately leading to cancer) characteristic of the non-exposed subjects is substantially increased from the moment the exposure starts.

We present and analyse here the age-mortality relationship of pleural mesothelioma as it is observed under endemic conditions in Karain. Deceased pleural-mesothelioma cases from the beginning of 1970 to the end of 1978 are included, subject to the following diagnostic criteria: (a) clinical and radiological evidence indicative of diffuse pleural mesothelioma, as in

TABLE I.—*Deaths from pleural mesothelioma, Karain,* 1970–78

| Years | | Age 20–29 | 30–39 | 40–49 | 50–59 | 60–69 | 70–79 | All ages |
|---|---|---|---|---|---|---|---|---|
| 1970–1974 | M | — | — | 6 | 3 | 4 | 1 | 14 |
| | F | — | 3 | 3 | 1 | 2 | 1 | 10 |
| 1975–1978 | M | 2 | 2 | 4 | 4 | 2 | — | 14 |
| | F | — | — | 3 | 5 | 4 | — | 12 |

previous descriptions (Baris *et al.*, 1978); (b) a clinical course leading to death in less than 2 years, irrespective of treatment; (c) histopathological diagnosis. For the 24 cases collected largely through a retrospective review of records between 1970 and 1974, no histopathological examination was available, whilst for the 26 cases directly investigated (1975–78) histopathological confirmation of mesothelioma was obtained in 19 (73%) specimens collected by thoracotomy (2), thoracoscopy (11) or punch biopsy (6). Table I gives the distribution of the 50 cases by age, sex and period of observation. During the total period 1970–78, a further 26 deaths due to causes other than mesothelioma were found among people aged 20 and over, bringing the total to 76 deaths (this figure is higher than the one derived from the local Health Centre Registry which we used for comparison between villages in our previous investigations (Baris *et al.*, 1981) and which was known to be affected by systematic underreporting).

A linear relationship between the logarithm of age and the logarithm of mortality rates was assumed and linear regressions were computed by a maximum-likelihood procedure (Baker & Nelder, 1978) using the natural log of age (mid-point of decennial classes) as the independent variable and the natural log of mortality rates as the dependent variable. For rates computation, population estimates for each age-sex group were derived from the local Health Centre Register. To reduce possible biases due to under-counting (of population and/or of cases) at older ages, only data for the ages 20–69 were used.

Due to the small numbers of cases on which the rates are based, the estimates of the exponent of age were very unstable when calculated within each subgroup of sex, period of observation and type of diagnostic ascertainment (with or without histological confirmation), but more stable results were obtained with the aggregate data. Results for the whole period of observation for males, females and both sexes together are shown in Table II and the Figure. The exponent of age providing the best fit to the observed mortality rates is 1·86 for males, 3·07 for females and 2·39 for both sexes combined. For all 3 lines there is no evidence of departure from log-linearity ($\chi^2$ values with 3 d.f.:

FIGURE.—Mesothelioma deaths in Karain, 1970–78 (age 20–69) (● Males, ○ Females).

TABLE II.—*Observed and expected deaths from pleural mesothelioma by age* (20–69) *and sex, Karain,* 1970–78

| | | 20–29 | 30–39 | 40–49 | 50–59 | 60–69 | All ages |
|---|---|---|---|---|---|---|---|
| Males | Pop | 428 | 218 | 344 | 363 | 163 | 1516 |
| | Obs | 2 | 2 | 10 | 7 | 6 | 27 |
| | Exp. | 2·6 | 2·5 | 6·4 | 9·6 | 5·9 | 27 |
| Females | Pop. | 505 | 330 | 521 | 307 | 190 | 1853 |
| | Obs. | 0 | 3 | 6 | 6 | 6 | 21 |
| | Exp. | 0·9 | 1·7 | 5·7 | 6·3 | 6·4 | 21 |
| Both sexes combined | Pop. | 933 | 548 | 865 | 670 | 353 | 3369 |
| | Obs. | 2 | 5 | 16 | 13 | 12 | 48 |
| | Exp. | 3·3 | 4·4 | 12·5 | 15·6 | 12·2 | 48 |

Pop. = total population (person-years);
Obs. = observed deaths;
Exp. = expected deaths, derived by means of the fitted equations:
log rate (per person-year) $= -11{\cdot}08 + 1{\cdot}86$ log age for males; log rate $= -16{\cdot}18 + 3{\cdot}07$ log age for females; log rate $= -13{\cdot}32 + 2{\cdot}39$ log age for both sexes combined.

2·9 for males; 2·7 for females; 8·5 for both sexes combined, with 8 d.f.). The 95%-confidence limits of these estimates are $\pm 1{\cdot}4$ for males, $\pm 1{\cdot}8$ for females and $\pm 1{\cdot}1$ for both sexes combined.

These cross-sectional estimates may be biased downwards by errors in age attribution of population estimates, secular changes in exposure, and age-related under-reporting of cases. However, none of them differs significantly from the estimated exponent of time since first exposure based on mesothelioma-mortality rates among North American insulation workers ($3{\cdot}2 \pm 0{\cdot}7$), or the similar exponent of age in the unexpected Los Angeles population (Peto *et al.*, 1982*a*, 1982*b*). The two related hypotheses proposed by these authors are thus consistent with these data pertaining to a general population (Karain) with a uniquely high occurrence of pleural mesothelioma: (i) that the mesothelioma pathogenic process follows the same sequence of cellular steps whatever the circumstances, though the type and intensity of carcinogenic exposure determines the rates at which these steps occur; (ii) that, in situations where no occupational exposure to asbestos or to other agents is recognized (the general population in Los Angeles and in Karain) the same mesothelioma-induction process begins at or soon after birth. This is supported by the fact that a similar power relationship between mortality and time holds, provided time is taken as time since first exposure (and not age or duration of exposure) in industrial cohorts exposed to asbestos dust, and as time since birth (*i.e.* age) in situations with no recognized exposure or where exposure begins at birth.

### REFERENCES

BAKER, R. J. & NELDER, J. A. (1978) *Generalised Linear Interactive Modelling.* Oxford: Numerical Algorithms Group.

BARIS, Y. I., SAHIN, A. A., OZESMI, M. & 5 others. (1978) An outbreak of pleural mesothelioma and chronic fibrosing pleurisy in the village Karain/Ürgüp in Anatolia. *Thorax,* **33,** 181.

BARIS, Y. I., SARACCI, R., SIMONATO, L., SKIDMORE, J. W. & ARTVINLI, M. (1981) Malignant mesothelioma and radiological chest abnormalities in two villages in Central Turkey. *Lancet,* i, 984.

DOLL, R. (1971) The age distribution of cancer: Implications for models of carcinogenesis. *J. R. Stat. Soc. Series A,* **134,** 133.

PETO, J., SEIDMAN, H. & SELIKOFF, I. J. (1982*a*) Mesothelioma mortality in asbestos workers: implications for models of carcinogenesis and risk assessment. *Br. J. Cancer,* **45,** 124.

PETO, J., HENDERSON, B. E. & PIKE, M. C. (1982*b*) Trends in mesothelioma incidence in the U.S. and the forecast epidemic due to asbestos exposure during World War II. In *Quantitation of Occupational Cancer.* Banbury Report No. 9, Cold Spring Harbour.

SARACCI, R. (1980) Epidemiology of groups exposed to other mineral fibres. In *Biological Effects of Mineral Fibres.* Ed. Wagner. Lyon: IARC Sci. Publ. No. 30. Vol. 2, p. 951.